# Formation of an ion-free crystalline carbon nitride and its reversible intercalation with ionic species and molecular water†
†Electronic supplementary information (ESI) available. See DOI: 10.1039/c8sc05232h


**DOI:** 10.1039/c8sc05232h

**Published:** 2018-12-21

**Authors:** Theo M. Suter, Thomas S. Miller, Jeremy K. Cockcroft, Abil E. Aliev, Martin C. Wilding, Andrea Sella, Furio Corà, Christopher A. Howard, Paul F. McMillan

**Affiliations:** a Department of Chemistry , University College London , Christopher Ingold Building, 20 Gordon Street , WC1H 0AJ , London , UK . Email: p.f.mcmillan@ucl.ac.uk; b Electrochemical Innovation Lab , Department of Chemical Engineering , University College London , Torrington Place , London WC1E 7JE , UK . Email: t.miller@ucl.ac.uk; c Materials and Engineering Research Institute , Sheffield Hallam University , City Campus , Howard Street , Sheffield , S1 1WB , UK; d Department of Physics & Astronomy and London Centre for Nanotechnology , University College London , Gower Street , London WC1E 6BT , UK . Email: c.howard@ucl.ac.uk

## Abstract

The development of processes to tune the properties of materials is essential for the progression of next-generation technologies for catalysis, optoelectronics and sustainability including energy harvesting and conversion. Layered carbon nitrides have also been identified as of significant interest within these fields of application. However, most carbon nitride materials studied to date have poor crystallinity and therefore their properties cannot be readily controlled or easily related to their molecular level or nanoscale structures. Here we report a process for forming a range of crystalline layered carbon nitrides with polytriazine imide (PTI) structures that can be interconverted by simple ion exchange processes, permitting the tunability of their optoelectronic and chemical properties. Notable outcomes of our work are (a) the creation of a crystalline, guest-ion-free PTI compound that (b) can be re-intercalated with ions or molecules using “soft chemistry” approaches. This includes the intercalation of HCl, demonstrating a new ambient pressure route to the layered PTI·*x*HCl material that was previously only available by a high-pressure-high-temperature route (c). Our work also shows (d) that the intercalant-free (IF-) PTI material spontaneously absorbs up to 10 weight% H_2_O from the ambient atmosphere and that this process is reversible, leading to potential applications for membranes and water capture in dry environments.

## Abstract 2

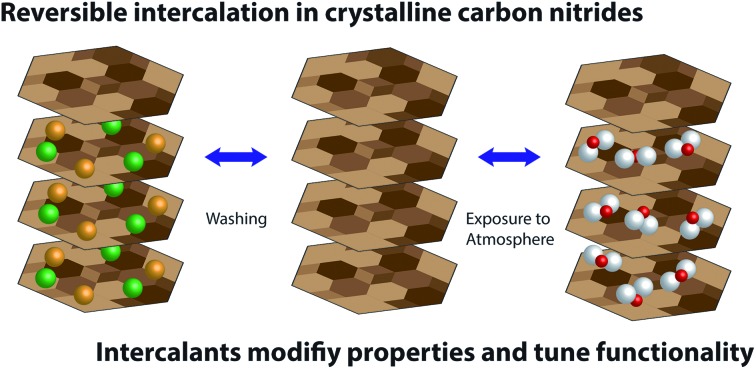
Crystalline layered carbon nitrides can be inter-converted by simple ion exchange process allowing their properties to be tuned.

## Introduction

Intercalation compounds are formed when atomic or molecular guest species are inserted between sheets of a layered host lattice.^1^–^3^ The process has widespread importance across fields ranging from battery technology and catalysis to filtration membranes and sequestration of molecular species from the atmosphere.^4^–^6^ Perhaps the best known host material is graphite, which can be readily and reversibly intercalated with Li^+^ ions. This property leads to its widespread use as the anode for most lithium ion batteries (LIB).^6^,^7^ Many other species can also be intercalated into graphite,^3^ as well as into other layered materials such as transition metal dichalcogenides,^8^,^9^ providing scope for tuning the properties and functionality of these layered phases and giving rise to new materials with technological applications. For example, the bandgap and electrical conductivity of a material can be modified by intercalation, due to charge transfer between the host layers and intercalant species.^2^,^10^ It has also been demonstrated that intercalation of ions can lead to convenient routes to liquid-phase exfoliation of layered materials, while maintaining the 2D crystallinity of the initial layers.^11^–^14^

Within layered materials the intralayer bonding is significantly stronger than that between the layers, giving rise to highly anisotropic properties. Such a bonding arrangement facilitates the intercalation process as the interlayer (out-of-plane) spacing expands or contracts readily as a function of the insertion or removal of intercalant species. Intercalation events can therefore be detected by monitoring changes in the unit cell dimensions, particularly the lattice parameter normal to the layers, using X-ray diffraction (XRD).^3^ The lattice expansion typically occurs without breaking covalent bonds within the layers and thus does not damage the host framework.^15^,^16^ Intercalation processes in layered materials can either occur spontaneously or be driven externally, as a function of chemical potential or electrochemical driving forces, in solid–gas or solid–liquid systems.^16^,^17^

**Fig. 1:**
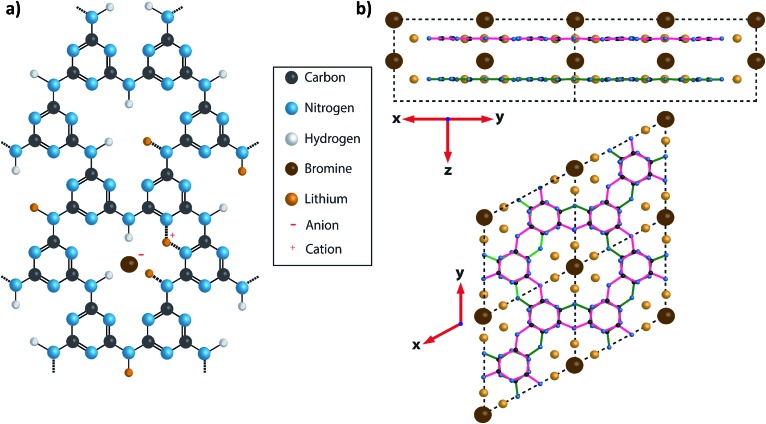
Diagram showing the structure of PTI·LiBr. (a) A charge balanced structure of PTI·LiBr showing the position of the Br^–^ anions located between the layers and the (assumed) positions of Li^+^ cations, that either partially replace H^+^ bound to the bridging imide units or between two adjacent triazine rings.^26^ In the present study the Br site occupancy was found to be approximately 50%. (b) A diagram of PTI·LiBr showing the layer stacking arrangement, with Br^–^ ions intercalated between the layers. The layers are arranged such that the triazine rings in successive layers are directly aligned above each other, but with bridging amine groups on alternating sides of the triazine ring in adjacent layers. This is termed the AA′ stacking motif.

Carbon nitrides, with N : C ratios > 1, form an important class of wide-gap semiconductors that are attracting increasing interest, most notably for their visible light absorption and photocatalytic properties.^13^,^18^–^20^ Polytriazine imide (PTI) carbon nitride structures are constructed from triazine (C_3_N_3_) rings, bridged by sp^2^ N–H groups. They form layers with C_6_N_9_H_3_ stoichiometry that resemble graphitic sheets, but contain regular structural voids (Fig. 1a and b). The crystalline sheets are typically stacked either in an AA′ fashion, with the triazine rings lying directly above and below each other (Fig. 1b), or as an AB motif where the void lies directly below and above the triazine rings of adjacent layers. The first of these crystalline PTI layered materials to be characterized was formed by a reaction between melamine and cyanuric chloride under high pressure (0.5–3 GPa) and high temperature (500 °C) conditions.^21^,^22^ The resulting compound was found to contain an additional HCl component incorporated within its structure, with Cl^–^ ions located approximately in the centre of the C_12_N_12_ voids within the carbon nitride plane, and the extra H^+^ protonating one of the surrounding triazine ring units.^21^,^22^ This material is referred to here as PTI·HCl.

An alternative synthesis route to related crystalline PTI materials was presented by M. Bojdys *et al.*^23^ who carried out a condensation reaction from dicyandiamide (DCDA, C_2_N_4_H_4_) in a molten eutectic LiCl/KCl or LiBr/KBr salt mixture at 600 °C. Although this original study suggested that the layers contained polyheptazine structural units, later work by Wirnhier *et al.* and others showed that the carbon nitride sheets were based on the same polytriazine imide motif as found for the PTI·HCl compound.^23^–^26^ The molten salt synthesis process produced PTI·LiCl or PTI·LiBr structures containing Cl^–^ or Br^–^ ions intercalated between the PTI layers (Fig. 1), along with Li^+^ ions either partly replacing H^+^ attached to the carbon nitride planes, or located between the layers (Fig. 1a).^23^–^26^ Chong *et al.* discussed the effect of anion size on the interlayer separation that determines the gallery height,^24^ and also noted that the Br content was lower than the fully intercalated theoretical stoichiometry of C_12_N_18_H_4_Li_4_Br_2_.^24^

Recent attention has focused especially on the photocatalytic activity of layered carbon nitride materials, leading to water splitting as well as other photocatalytic processes important for energy and sustainability applications.^27^–^32^ These compounds typically exhibit colours in the yellow-brown range, indicating visible as well as UV light absorption, with bandgaps stated to occur in the 2.4–2.7 eV range. It has been shown by McDermott *et al.* that tuning the Li^+^, H^+^ and other intercalated ion site occupancies can be used to alter the optical bandgap and thus influence the photocatalytic activity.^10^

Here we describe a method to fully remove the intercalated ions from PTI materials to create an ‘intercalant-free’ (IF-) PTI compound, and then replace them using ‘soft chemistry’ techniques with different ionic species. This points the way to the creation of a new family of crystalline layered PTI materials with tunable optoelectronic and chemical properties. Our IF-PTI phase constitutes the first substantiated example of a crystalline layered PTI-structured ion-free carbon nitride compound, with stoichiometry C_2_N_3_H. We also observe that this material spontaneously and reversibly absorbs H_2_O from the ambient atmosphere.

## Results and discussion

**Fig. 2:**
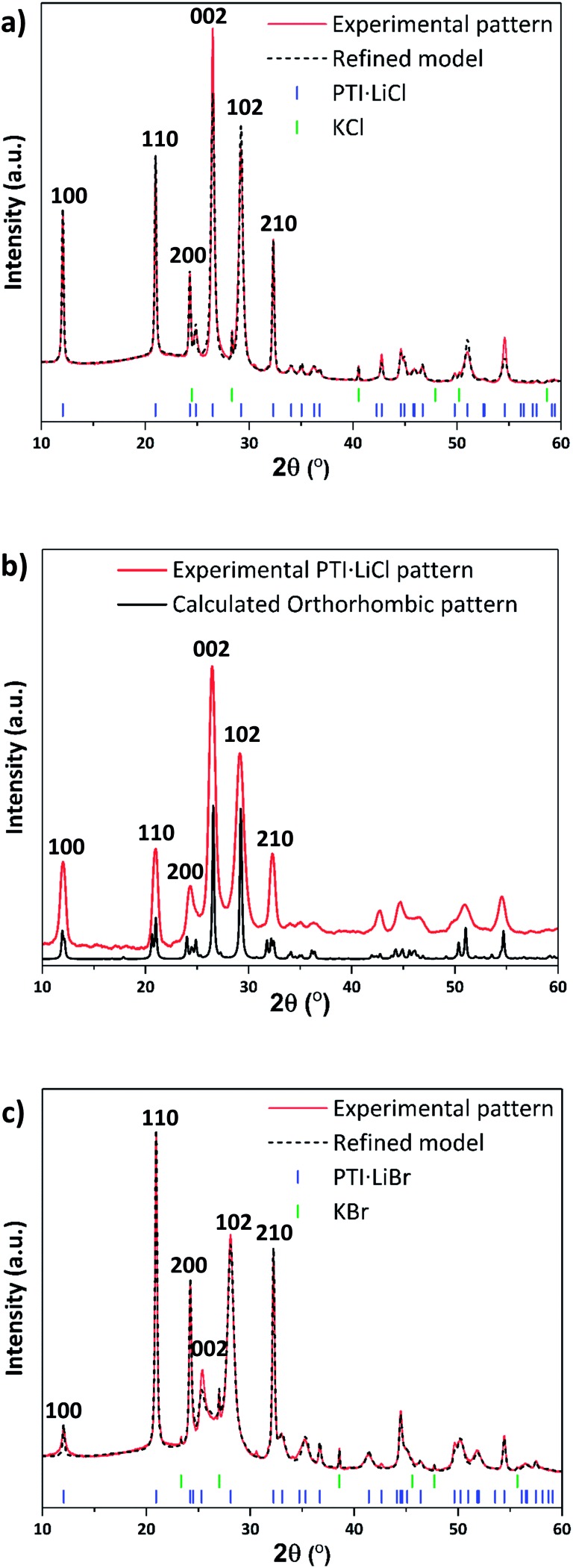
PXRD patterns and refinements of PTI·LiCl and PTI·LiBr. (a) PXRD of PTI·LiCl with refined pattern based on hexagonal structure. (b) PXRD of PTI·LiCl compared with calculated pattern using an orthorhombic unit cell. (c) PXRD of PTI·LiBr with refined model based on the hexagonal unit cell.

The starting compounds for our study were crystalline PTI·LiCl and PTI·LiBr materials prepared *via* the previously published molten salt synthesis approach (see ESI,† for further details).^24^,^26^ Powder X-ray diffraction (PXRD) data of these materials were analysed and compared with predicted patterns using Mercury^33^,^34^ and Rietveld refinement (Rietica^35^) software (Fig. 2a–c). A hexagonal unit cell with space group *P*6_3_*cm* was indicated by Wirnhier *et al.*^26^ and Chong *et al.*^24^ for both structures. For each of these materials our data (both in terms of peak positions and relative intensities) closely match these hexagonal structure models, with *R*_wp_-factors refined as 6.8% and 5.6%, for PTI·LiCl and PTI·LiBr respectively. However, from a combination of solid state nuclear magnetic resonance (NMR) and pair distribution function (PDF) analysis of total X-ray scattering data, Mesch *et al.* indicated a more precise location of Li^+^ and H positions within the PTI·LiCl structure, proposing that the structure could be better modelled using an orthorhombic unit cell with *P*2_1_2_1_2_1_ symmetry.^25^ In Fig. 2b we show our PXRD data for PTI·LiCl compared with the pattern calculated for this orthorhombic cell model. The symmetry change from *P*6_3_*cm* to *P*2_1_2_1_2_1_, combined with the larger unit cell size, would result in splitting of the (100), (110) and (210) reflections (defined relative to the hexagonal cell), which we do not observe experimentally. Likewise, several additional peaks that would be expected for the orthorhombic phase are absent. We did attempt to refine our PTI·LiCl data within the suggested orthorhombic space group and structure, but the refinement consistently returned to the hexagonal solution. We have therefore used the hexagonal unit cell model to describe the PTI structures presented in this report.

The gallery height, defined as the distance between two adjacent carbon nitride layers, is readily extracted from the 2*θ* or *d*_*hkl*_ value of the (002) reflection.^36^ This peak changes from 26.5° 2*θ* (3.36 Å) for PTI·LiCl to 25.2° 2*θ* (3.52 Å) in PTI·LiBr, representing the 0.16 Å change in interlayer spacing expected for intercalation of the larger halide ion (ionic radii: 1.81 Å for Cl^–^, 1.96 Å for Br^–^).^24^,^37^ We note that the nature and degree of occupancy of the halide ion site has a significant effect on the PXRD patterns of PTI materials, because the high-Z intercalant species (Cl^–^ and Br^–^) dominate the X-ray scattering intensity over the lighter elements (C, N, H) that determine the layered structures.^19^ We also note that some broadening occurs for reflections with a non-zero “*l*” Miller index component compared with *hk*0 reflections in the PTI·LiCl and PTI·LiBr diffraction patterns. Such differences in peak broadening between in-plane and between-plane reflections is commonly observed among layered materials that can exhibit different degrees of structural order within and between the planes.^36^,^38^

### Ionic de-intercalation studies

**Fig. 3:**
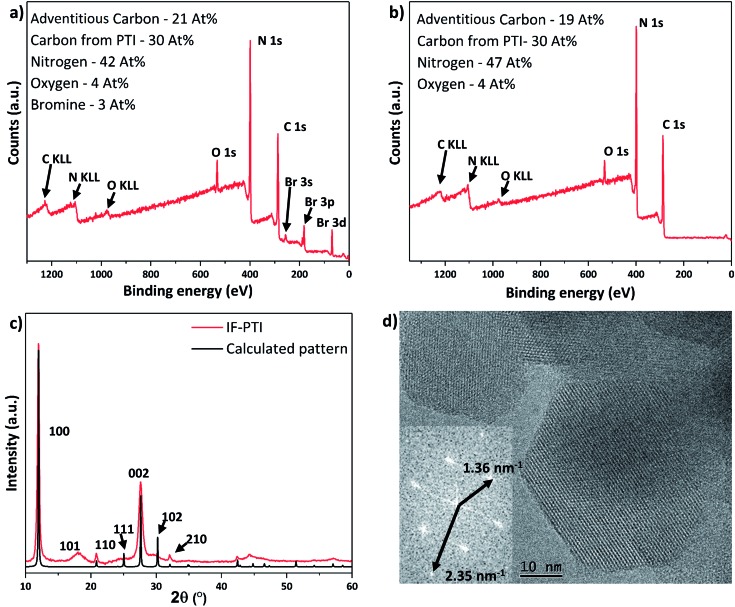
XPS and PXRD data for PTI·LiBr and IF-PTI. Elemental compositions are given in atomic percent. The C content from PTI is reported after removal of the contribution from adventitious signals. (a) XPS survey scan of PTI·LiBr showing significant C, N and Br content, along with the presence of a small percentage of O impurities, presumably due to surface contamination. (b) XPS survey scan of IF-PTI showing similar C, N and O content, but with the Br signal notably absent. (c) PXRD pattern of IF-PTI overlaid with calculated pattern based on the PTI·LiBr structure (assuming AA′ stacking with a smaller interlayer spacing), demonstrating the significantly smaller interlayer spacing. (d) TEM image of hexagonal crystallites of IF-PTI with a Fourier transformed pattern showing the characteristic in-plane distances of the 100 and 110 reflections for PTI (for IF-PTI it was found *a* = *b* = 8.51 Å, *c* = 6.58 Å).

Li^+^, Br^–^ and Cl^–^ ions were removed from the PTI·LiCl and PTI·LiBr layered structures using continuous Soxhlet extraction in deionized water, typically for 20 days for complete extraction (see ESI† for further details). The resulting solids were dried under reduced pressure and then extensively examined using PXRD, X-ray photoelectron spectroscopy (XPS) and high resolution transmission electron microscopy (HRTEM) to verify their structure. Both PTI·LiCl and PTI·LiBr starting materials yielded the same intercalant-free (IF-) PTI material, consisting only of the crystalline CNH layered backbone, established by comparing the PXRD and XPS data (Fig. 2 and 3), with theoretical composition C_6_N_9_H_3_ (or C_2_N_3_H). Neither material contained any detectable residual halide component. Quantification of the Li content was achieved using quantitative solid state ^7^Li MAS NMR spectroscopy, using the integrated intensity of the central isotropic peak and spinning sidebands. This showed that only 1 ± 0.1% Li remained in the IF-PTI samples compared with the starting compounds (see ESI† for details). Our XPS data showed that the C : N ratio remained constant, and that there was no observable change in the O impurity content of the materials (Fig. 3a and b). Comparing the fitted XPS C 1s and N 1s peaks from each structure (Fig. S5–S7†) demonstrated that the chemical environments of these elements within the carbon nitride layers remained unchanged. These data substantiate that Soxhlet extraction involving continuous washing of the PTI·LiCl and PTI·LiBr starting materials in water results in the production of a new intercalant-free (IF-) form of crystalline layered PTI, with layer composition C_6_N_9_H_3_ (C_2_N_3_H). The stoichiometry established from elemental analysis of PTI·LiBr and IF-PTI obtained *via* two different methods (XPS and combustion analysis) is provided in Table 1. These analyses show that the C to N ratio is not affected by the washing procedure. However, the H content is slightly higher in the IF-PTI material due to replacement of Li^+^ ions in the structure by H^+^ ions derived from the aqueous extraction medium. This is borne out by pH measurements that showed the water reservoir in the Soxhlet reactor to have changed from a starting value of ∼7 to close to 10. This increase in pH can be attributed to the exchange of Li^+^ from the PTI·LiCl or PTI·LiBr materials with H^+^ derived from the water, resulting in an increase in OH^–^ concentration within the aqueous phase.^39^

**Table 1:** Elemental composition from XPS, combustion analysis and NMR for PTI·LiBr and IF-PTI

	XPS	Combustion analysis	^7^Li content from NMR
PTI·LiBr	C_12_N_16.8_Br_1.0_	C_12_N_16.6_H_10.2_Br_1.3_	C_12_N_16.6_H_10.2_Li_1.9_Br_1.3_
IF-PTI	C_12_N_18.8_Br_0.0_	C_12_N_16.2_H_13.3_Br_0.05_	C_12_N_16.2_H_13.3_Li_0.015_Br_0.05_

The PXRD pattern of IF-PTI (Fig. 3c) could be analysed using the same in-plane lattice parameters as those established for PTI·LiBr, indicating that the internal structure and bonding within the PTI layers were not affected by the washing and de-intercalation process (Fig. 2c). However, all peaks with an out-of-plane *hkl* component shifted to higher 2*θ* values as a result of the substantially reduced interlayer spacing from 3.52 Å to 3.23 Å (Fig. 3c). These results show that the base gallery height of crystalline IF-PTI is even smaller than that of graphite (3.35 Å),^40^ perhaps because the localized partial charges between carbon and nitrogen result in interlayer attraction.^32^ In all the PTI materials investigated in this study we found that reflections with a non-zero (*l*) component were broadened compared with (*hk*0) reflections (Fig. 2a and c). This anisotropic peak broadening was more evident in the PXRD pattern for IF-PTI. The de-intercalation process results in large changes in the relative intensities of different reflections in the PXRD patterns, because of the removal of the high-Z elements with their large contribution to the X-ray structure factor and diffraction intensities. For example, this results in the significantly stronger (100) reflection observed in the IF-PTI PXRD pattern (Fig. 3c) compared with that for PTI·LiBr (Fig. 2c). The combination of broadening and shifts in peak position for reflections with an (*l*) component, along with the change in relative peak intensity, causes some of the characteristic peaks from the starting PTI·LiBr phase (such the (102) and (111) reflections) to be reduced in intensity so that they fall below the detection sensitivity of the instrument. We also observed an additional reflection for IF-PTI at ∼18° 2*θ*, that can be explained by the appearance of the (101) reflection for the hexagonal unit cell, which is not observed for PTI·LiBr or PTI·LiCl. This peak appears because of the change of symmetry that occurs as the layer stacking pattern changes from AA′ in which the voids ‘line up’ along the *c* axis, to AB with a triazine ring located directly above and below each void. This change in layer stacking motif is clearly seen for the PTI·HCl material (Fig. 5c), and it can be explained by the loss of anchoring between layers by the intercalated ions, as well as by the different relative sizes of the LiBr/LiCl *vs.* HCl intercalants that have been removed or exchanged.

The hexagonal crystallites observed in TEM images of IF-PTI are identical to those reported for PTI·LiBr and PTI·LiCl materials prepared by similar synthesis routes (Fig. 3d).^23^,^26^ Our TEM studies did not reveal any detectable increase in defect density of the crystallites following the Soxhlet extraction procedure. The in-plane characteristic distances measured from Fourier transforms of our TEM images remained unchanged following washing, confirming that in-plane ordering within the carbon nitride layers was not affected by the de-intercalation process. The TEM images combined with the PXRD, elemental analysis and XPS data show that the de-intercalation procedure removed the Li^+^ and halide intercalant species without any structural damage to the covalently bonded PTI framework. The resulting solids containing only carbon, nitrogen and hydrogen with a layer composition C_6_N_9_H_3_ (C_2_N_3_H) represent a new intercalant-free (IF-) PTI phase, containing layers of triazine rings bridged by secondary imido (–NH–) groups.

### Reversible intercalation of H_2_O into IF-PTI under ambient atmospheric conditions

**Fig. 4:**
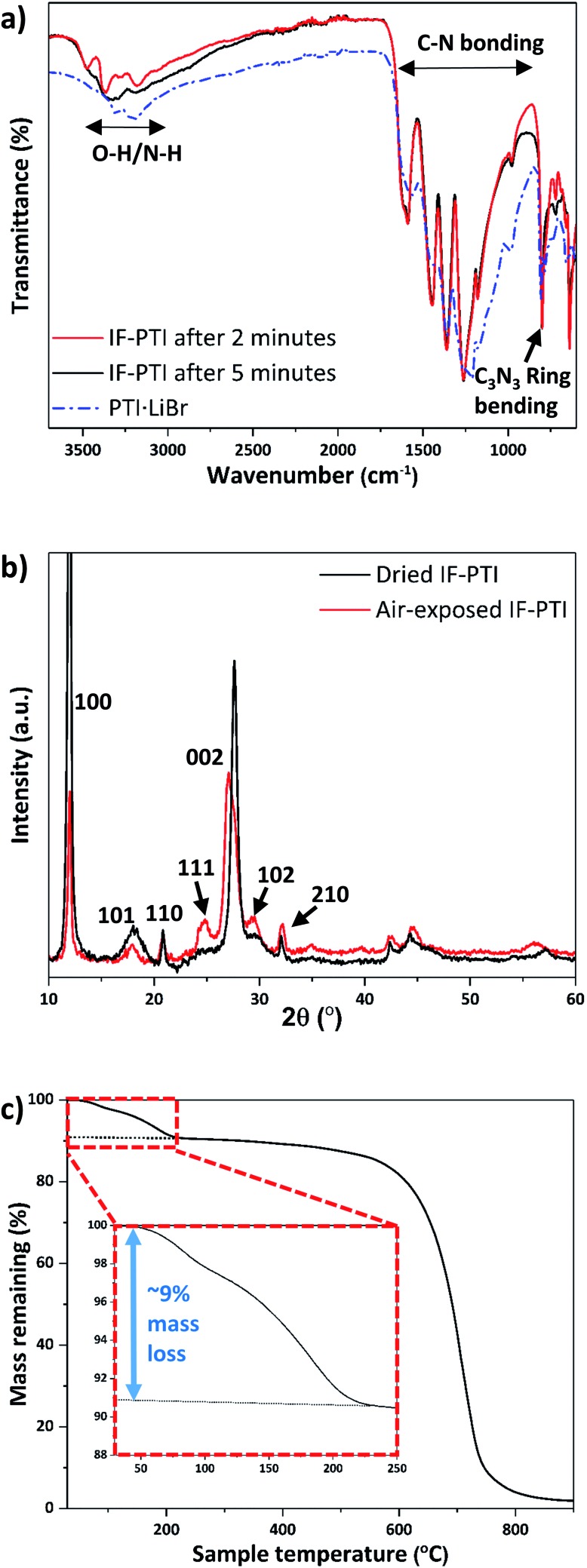
IR, PXRD and TGA results for IF-PTI before and after exposure to laboratory air, demonstrating spontaneous uptake of atmospheric moisture by intercalation of H_2_O. (a) FTIR spectra of PTI·LiBr and IF-PTI immediately after its first exposure to the air atmosphere, followed by a spectrum taken after 5 minutes of exposure. (b) PXRD patterns of “wet” *vs.* “dry” IF-PTI, showing the changes due to H_2_O incorporation between the layers. (c) TGA of IF-PTI after exposure to atmospheric moisture, demonstrating incorporation of ∼9 wt% H_2_O within the structure (inset).

We examined the various PTI materials prepared in this study using Fourier-transform infrared (FTIR) spectroscopy. The IF-PTI samples showed C–N bonding peaks between 1200–1600 cm^–1^ at the same positions as for the PTI·LiCl or PTI·LiBr starting materials, however the peaks were significantly sharpened following the washing procedure (Fig. 4a). A number of bands were observed in the 2700–3700 cm^–1^ region for freshly dried IF-PTI (see methods, ESI†), but they quickly exhibited a very significant, and unexpected, broadening after only a few minutes of exposure to ambient atmospheric conditions (Fig. 4a). These peaks occur in a region that is typically assigned to N–H stretching vibrations of the PTI materials,^21^,^22^,^24^–^26^ however they could also be assigned to hydrogen-bonded OH or H_2_O units present within the layered structures. The PXRD patterns of dry IF-PTI likewise showed a change after exposure to air (Fig. 4b). While the in-plane *hk*0 peaks did not shift in position, there was a clear change in any peak with an *l* component, including the main (002) reflection that shifted to a smaller 2*θ* value indicating a change in interlayer spacing from 3.23 Å to 3.29 Å. This was accompanied by a change in the relative peak intensities, resulting in the ‘emergence’ of peaks that were previously too weak to be observed above the background. We propose that these changes in the IR spectra and PXRD patterns are consistent with the occurrence of rapid and spontaneous intercalation of H_2_O molecules, which have been absorbed into the IF-PTI structure from the surrounding ambient atmosphere. Examination of our IF-PTI materials that had been exposed to the atmosphere using thermogravimetric analysis (TGA, Fig. 4c) showed an initial and atypical 9 wt% mass loss which occurred during heating between 80–200 °C, assigned to the loss of water. A second mass loss event occurred between 600 and 800 °C that is typically observed for carbon nitrides due to thermal decomposition of the C–N bonded layer structure.^41^,^42^ Our mass balance calculations (see ESI† for further details) indicate that the loss of one H_2_O molecule from each of the available void sites within the C_6_N_9_H_3_ compound would result in such a 9 wt% mass loss. When the air-exposed IF-PTI sample was heated to 150 °C under reduced pressure for several hours, the PXRD pattern returned to that observed initially for the freshly dried IF-PTI (Fig. 4b). This process could be repeated several times with no indication of any modification to the structure. We conclude that the exposure of IF-PTI to ambient air has resulted in spontaneous uptake and incorporation of atmospheric H_2_O within the interlayer sites, leading to formation of a previously unknown H_2_O-intercalated phase of PTI which we define as PTI·H_2_O. The H_2_O intercalation process occurs spontaneously within a relatively dry laboratory atmosphere, so the IF-PTI material could act as a desiccant or absorbent for atmospheric moisture, whereas the reversibility of the process suggests the potential for membrane or sequestration-release applications.

### Ionic re-intercalation processes

**Fig. 5:**
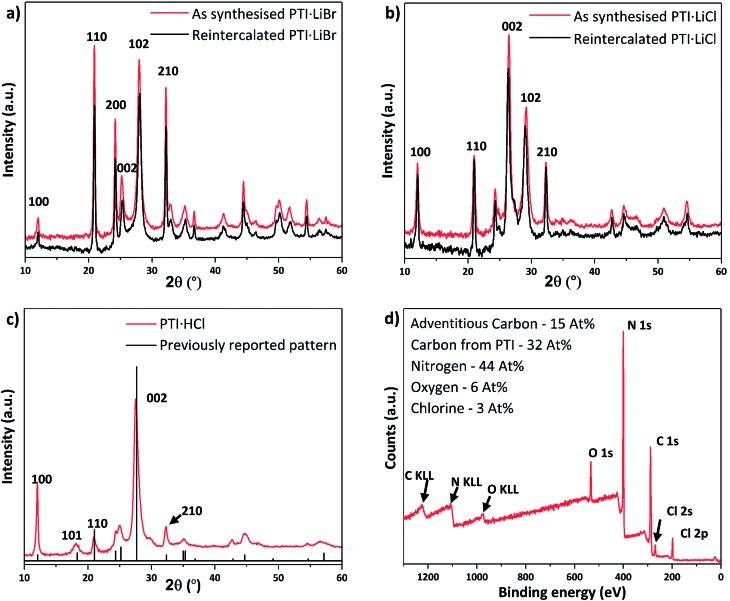
PXRD patterns and XPS spectrum of re-intercalated PTI structures. (a) Experimental PXRD pattern of PTI·LiBr and RI-PTI·LiBr, showing an exact overlap between the two datasets. (b) PXRD pattern of PTI·LiCl and RI-PTI·LiCl, showing a clear overlap between data obtained for material synthesised *via* the molten salt route and that produced by re-intercalation from IF-PTI. (c) The PXRD pattern obtained from re-intercalated PTI·HCl overlaid with reported peak intensities and positions from previous materials obtained *via* high-*P*,*T* synthesis. (d) XPS survey scan of our RI-PTI·HCl phase, showing an increase in intercalated Cl content.

Following the initial deintercalation step, samples of dry IF-PTI were then re-exposed to a molten salt (LiBr : KBr) mix in a sealed ampoule under reduced pressure (see ESI† for details).^1^ This reaction was designed to study re-intercalation of the Li^+^ containing salt back into the IF-PTI framework, to re-form the initial PTI·LiBr intercalated compound and thus demonstrate that the de-intercalation/re-intercalation process could occur without damaging the carbon nitride backbone. Similar approaches have been applied to create a wide range of graphite intercalated compounds (GICs).^1^,^43^ PXRD results show that the re-intercalated (RI-PTI·LiBr) is structurally identical with the starting PTI·LiBr compounds (Fig. 5a). We further investigated the procedure by exposing IF-PTI to a LiCl : KCl molten salt mixture, and found that the resulting PXRD pattern corresponded exactly with that of crystalline PTI·LiCl, obtained using molten salt synthesis (Fig. 5b). These results demonstrate that the intercalation processes are entirely reversible, leading to the possibility of creating new PTI phases depending on choice of molten salt used for re-intercalation.

After refluxing IF-PTI with 5 M HCl for 4 days we observed formation of a PTI·HCl phase. Lin *et al.* have attempted to create this material at atmospheric pressure, but this resulted in a material that is inconsistent with the original PTI·HCl created using high-*P*,*T* reactions from molecular precursors.^21^,^22^,^44^ PTI·HCl has been predicted to exhibit improved photocatalytic hydrogen evolution properties due to its optimized band gap, so that our potentially scalable method of producing it at ambient conditions *via* re-intercalation of IF-PTI should prove useful.^10^ Our XPS data demonstrated a substantial increase in the Cl content, without any changes occurring in the C/N ratio (or O content) (Fig. 5c and d). The XPS data indicated a Cl^–^ site occupancy near 65%. The PXRD pattern for the RI-PTI·HCl phase is almost identical to data reported previously for materials formed by high-*P*,*T* synthesis, but with minor differences in the relative intensities of the (100), (110) and (210) reflections and a slight change in the position of the (002) interlayer reflection from 27.7° 2*θ* to 27.5°, associated with the lowered Cl site occupancy.^21^,^22^,^24^ The appearance of the (101) reflection at ∼18° 2*θ* demonstrates the change in layer stacking from AA′ to AB analogous to that observed for IF-PTI (Fig. 3c).

### UV-visible spectroscopy results

**Fig. 6:**
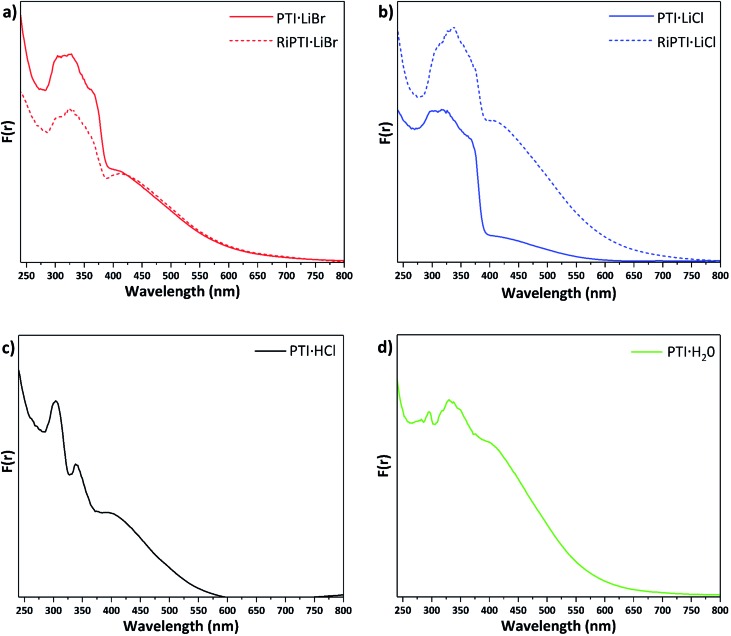
UV-visible absorption spectra presented in terms of the Kubelka–Munk function *F*(*r*) for different PTI materials (a) PTI·LiBr and RI-PTI·LiBr, (b) PTI·LiCl and RI-PTI·LiCl, (c) PTI·HCl (d) PTI·H_2_O prepared in this study.

Understanding and controlling variations in the electronic structure of layered carbon nitrides is essential to target optoelectronics applications. We measured UV-visible absorption spectra for the various PTI materials prepared in this study (Fig. 6). The spectra for PTI·LiBr and PTI·LiCl (Fig. 6a and b) are identical with previously reported data,^39^,^45^ although earlier data were only reported to 350/400 nm. The main absorption band, with a steep onset below 380 nm and maxima occurring near 310, 320 and 370 nm, is typically assigned to π–π* transitions of the carbon nitride framework.^31^,^46^ In PTI·LiCl the highest energy valence band states are expected to have a significant contribution from the chlorine 2p electrons,^10^,^46^ but because these atoms are spatially isolated from the carbon nitride layers they are not expected to play a significant role in photocatalysis and photoabsorption processes.^10^ The identical peak positions and band onsets observed for PTI·LiBr and PTI·LiCl compounds indicate that the halide ions do not play a significant role in defining the band gap properties.

The features at longer wavelength, beginning near 650 nm and culminating in a broad peak at ∼420 nm, have been attributed to n to π* transitions associated with lone pairs on N atoms in the triazine rings becoming excited into the π* orbitals.^31^,^46^ These transitions are forbidden for highly symmetrical planar layers, but become allowed as loss of symmetry and layer buckling occurs.^31^,^46^ The UV/Vis spectra of previously de-intercalated and then re-intercalated RI-PTI·LiCl and RI-PTI·LiBr samples (Fig. 6a and b) show the same π to π* transition bands, but both exhibit more pronounced n to π* absorption features, most likely due to minor structural distortions and layer asymmetries occurring as the Li^+^ and Cl^–^, Br^–^ ions were introduced during the re-intercalation process.

Unlike PTI·LiCl and PTI·LiBr samples, the spectrum of PTI·HCl shows two distinct maxima at 300 and 340 nm in its π–π* transition region, and the rise in absorption coefficient attributed to the bandgap onset is much less marked (Fig. 6c). However, the broad n–π* absorption occurs over a similar range. McDermott *et al.*^10^ suggested that partial replacement of H^+^ with Li^+^ at the imide positions should cause splitting to occur in the N valence band states at highest energy.^10^ This likely contributes to the complexity and broadening of the π–π* region for the PTI·LiCl and PTI·LiBr samples, which partially retain NH groups at the bridging imide sites. PTI·HCl contains no intercalated Li^+^ so there is no splitting of the N valence band states and less hybridization with Cl orbitals, hence the sharpness of the observed peaks. If the bandgap is taken as the onset of the first π–π* peak near 360 nm, then there is a slight increase in bandgap energy compared with ∼375–380 nm values observed for PTI·LiCl and PTI·LiBr (Fig. 6). The PTI·H_2_O sample shows a further evolution of the spectrum, with a weaker feature at 290 nm, a main broad π–π* peak occurring at ∼340 nm with its onset near 360 nm, and a broad n–π* absorption similar to the other materials (Fig. 6d). The observed strength of the n–π* absorption for this sample could be associated with incorporation of intercalated H_2_O molecules, resulting in loss of local symmetry, and enhanced intensity of the normally forbidden transition.

## Conclusion

We have substantiated a new intercalant-free crystalline carbon nitride phase with a polytriazine imide layered structure (IF-PTI). This is achieved without loss of layer ordering through extraction of the intercalated ions into aqueous solution using a low temperature process. The resulting IF-PTI material can then act as a reversible host material for a range of intercalates, introduced either from molten salts or from aqueous solutions. We have demonstrated the reversibility of the intercalation process to reproduce the starting PTI phases, as well as new compounds by the re-intercalation of other combinations of ion pairs, shown here for the H^+^, Li^+^, Cl^–^, Br^–^ set of intercalants, but could be extended to other intercalant combinations. The resulting changes in electronic structure as a function of layer separation and intercalant species can be useful for tuning the bandgap as well as other optoelectronic properties for specific applications. We also observed that the IF-PTI material spontaneously and reversibly absorbed H_2_O from the atmosphere, by intercalation of the water molecules into the layered PTI structure. The resulting compound constitutes a new carbon nitride layered compound, PTI·H_2_O. The intercalated water could be removed without damaging the PTI framework. Such rapid and reversible water intercalation could lead to new applications for these materials for water absorption/collection in dry environments, as well as potential membrane and filtering applications.

## Conflicts of interest

There are no conflicts to declare.

## Supplementary Material

Supplementary information
