# Supplementary material for: Formation of an ion-free crystalline carbon nitride and its reversible intercalation with ionic species and molecular water
Source: Chem Sci. 2018 Dec 21;10(8):2519–28. doi: 10.1039/c8sc05232h (PMC6385848; doi:10.1039/c8sc05232h)
Supplement: Supplementary file 1 [file SC-010-C8SC05232H-s001.pdf]

Electronic Supplementary Material (ESI) for Chemical Science.
This journal is © The Royal Society of Chemistry 2018

Supplementary information

Formation of an ion-free crystalline carbon nitride and its reversible intercalation with ionic species and molecular water

Theo M. Suter,[a, b] Thomas S. Miller,[b*] Jeremy K. Cockcroft, [a] Abil E. Aliev, [a] Martin C. Wilding, [a, c] Andrea Sella,[a] Furio Corà,[a] Christopher A. Howard,[d*] and Paul F. McMillan[a*]

[a] Department of Chemistry, University College London, Christopher Ingold Building, 20 Gordon Street, WC1H 0AJ, London, U.K.

[b] Electrochemical Innovation Lab, Department of Chemical Engineering, University College London, Torrington Place, London WC1E 7JE, UK.

[c] Materials and Engineering Research Institute, Sheffield Hallam University, City Campus, Howard Street, Sheffield, S1 1WB, UK.

[d] Department of Physics & Astronomy and London Centre for Nanotechnology, University College London, Gower Street, London WC1E 6BT, U.K.

*p.f.mcmillan@ucl.ac.uk; t.miller@ucl.ac.uk; c.howard@ucl.ac.uk

Synthesis of PTI·LiBr/PTI·LiCl

PTI·LiBr and PTI·LiCl were synthesised by a procedure based upon that first reported by Bojdys *et al.*[1] Dicyandiamide (2 g, Sigma Aldrich) and a eutectic mix of KBr (4.8 g, Alfa Aesar) and LiBr (5.2 g, Aldrich), or KCl (5.5 g, Aldrich) and LiCl (4.5g, Aldrich), were thoroughly ground together by hand under an N_2 atmosphere. This mix was packed into an alumina crucible and placed under an inert N_2 flow and heated in a tube furnace to 400 ^oC for 6 hours (10 ^oC min^{-1} ramp rate, Carbolite, MTF 12/38/250). The resultant solid mass was then placed in a glovebox and again ground by hand. Approximately 11.5 g of this powder was transferred, under argon, to a quartz ampoule (OD 23mm, ID 20mm, Multilab Ltd) that was evacuated to 10^{-6} mbar using a turbo pump (Leybold vacuum systems, PT70G compact). The tube was then flame-sealed to form an ampoule under static vacuum, placed in a chamber furnace (Carbolite, CWF11/13) and heated to 600 ˚C (ramp rate 10 ˚C min^{-1}) where it was held for 12 hours before being cooled to room temperature (ramp rate 10 ˚C min^{-1}). The solid PTI product was recovered by washing the solidified salt block multiple times with distilled water (Millipore) to remove the soluble salt component, followed by ethanol, before it was dried using a rotary evaporator.

De-intercalation of PTI·LiX to form IF-PTI

The intercalated ions were removed from the PTI·LiX compounds via Soxhlet extraction.[2] In each extraction ~1.5 g of PTI was loaded into a cellulose thimble (Whatman Cellulose extraction thimbles, OD 27 mm, ID 25 mm, length 80 mm) and was typically washed for 20 days with hot distilled water. The resulting intercalant-free (IF-) PTI was filtered, then washed several times with ethanol, before being dried at 150 ˚C at 10^{-2} mBar and stored under an inert atmosphere.

Re-intercalation in a eutectic salt melt

IF-PTI (180 mg) was added to a eutectic mix of either (KBr Alfa Aesar 0.96 g: 1.04 g LiBr Aldrich) or (KCl Aldrich 1.1g : 0.9 g LiCl Aldrich). These were ground together and sealed inside a quartz ampoule and under static 10^{-6} mbar pressure. This was

then heated to a temperature above the melting point of the eutectic salt mixture and held for a period of time (up to 12 hours). The recovered block was then washed multiple times with distilled water (Millipore), then ethanol and dried via rotary evaporation.

Liquid phase re-intercalation

IF-PTI (100 mg) was placed in 5 M HCl solution (75 ml, Sigma Aldrich) and refluxed for 4 days. The resulting powder was recovered by filtration and washed several times with distilled water (Millipore) and then ethanol, before being dried at 150 ˚C at 10^{-1} mBar and stored under an inert atmosphere.

Materials characterisation techniques

Field emission scanning electron microscopy (FE-SEM) images were taken using a JEOL JSM-6700F system and accelerating voltage of 5 kV. Samples were mounted on conductive carbon tape and gold coated before imaging. Transmission electron microscopy (TEM) studies were performed using a JEOL JEM-2100 TEM instrument at an acceleration voltage of 200 kV. Samples were prepared by sonication in methanol, followed by dropping the resultant suspension onto 300 mesh gold TEM grids with a holey carbon film (Agar Scientific). Additional high resolution TEM (HRTEM) images were collected using a FEI Titan 60–300 instrument with 80 kV accelerating voltage. TEM images were analysed using the software Digital Micrograph (Gatan). XPS measurements were performed using a Thermo Scientific spectrometer using monochromated Al K_{α} radiation. Samples were mounted using conductive carbon tape and a charge compensating flood-gun was utilised during measurements due to the semiconductive nature of the PTI. Powder XRD patterns were measured on a Stoe Stadi-P diffractometer using Cu K_{α} radiation equipped with a Mythen 1k detector. Samples were filled in either 0.5 or 0.7 mm X-ray glass capillaries and rotated during the measurement. Fourier transform infra-red (FTIR) spectra were obtained using a Bruker IRscope attached to an IFS-66v bench in transmittance mode. ATR-FTIR spectra were obtained using a Bruker Alfa instrument.

Samples were sent to University of Sheffield for CNH elemental analysis using a Vario MICRO cube elemental analyzer (Elementar). TGA was run on a Netszch TGA instrument. The material was loaded into an alumina crucible (~5 mg), which was heated under He at 5 ˚C/min from room temperature to 750 ˚C. Solid-state NMR experiments were carried out on a Bruker Avance 300 spectrometer with a 7.05 T wide-bore magnet at ambient probe temperature. High-resolution solid-state ^{7}Li spectra were recorded at 116.6 MHz using a standard Bruker 4 mm double-resonance magic-angle spinning (MAS) probe (Fig. 1). Powdered solid materials were packed into zirconia rotors of 4 mm external diameter and spun at the MAS frequency of 8 kHz with stability better than ±3 Hz. To suppress acoustic ringing effect for better spectral baseline, a standard *aring* sequence from the Bruker pulse program library was used. For quantitative estimates, a recycle delay of 60 s was used (total acquisition time 20 hours). The ^{7}Li chemical shifts were referenced indirectly to Li^{+} using a dilute aqueous solution of LiCl.[3]

Compositional characterization

The chemical compositions were determined using a combination of bulk chemical analysis and X-ray photoelectron spectroscopy (XPS) techniques (Figure 3,4, S5,S6,S7). Previous studies of crystalline PTI·LiCl have reported compositions extending up to $C_{12}N_{17.5}H_{6.3}Cl_{1.5}Li_{3.2}$,[4] corresponding to a halide ion site occupancy of 75%, whereas our XPS analyses indicate an interlayer site occupancy of 95%. Likewise, Chong *et al* found a composition for the PTI·LiBr compound near $C_{12}N_{17.8}H_{0.97}$ $Li_{1.0}Br_{0.2}$,[5] indicating a Br^{-} site occupancy of 10%, whereas our materials showed occupancies extending up to 48% of the available interlayer sites. The lowered anion site content observed in previous studies could have been caused by post-synthesis washing procedures that we have shown can partly remove the intercalated ionic species.

Water absorption calculation

A single unit cell of IF-PTI has the stoichiometry $C_{12}N_{18}H_6$, with a total mass 402 a.m.u. contains two interlayer void spaces in which the Cl^-/Br^- ions were previously intercalated per C_{12} unit. These could be re-filled with H_2O molecules to give a water re-intercalated composition $C_{12}N_{18}H_6{\cdot}(H_2O)_2$, with mass 438 a.m.u.. Subsequent loss of the H_2O component during heating would cause a mass loss of 8.2%, which corresponds closely with the value observed by TGA (~9%). No such mass loss is observed for PTI·LiBr or PTI·LiCl across the same temperature range.

Lithium content determination

Determining the content of the light element Li is notoriously difficult using combustion or XPS analysis. We determined the lithium content of PTI materials prepared in this study using ^{7}Li MAS NMR spectroscopy. The total integral intensity of the ^{7}Li signal (i.e., the sum of the integral intensities of the isotropic peak and spinning sidebands) was first determined for a known mass of LiBr, to provide a standard measure of integral NMR intensity per mole of Li present. This was then compared with the NMR signal intensity observed for different PTI samples. The ^{7}Li intensity for the PTI·LiBr phase was 3 times lower than for LiBr, consistent with the composition $C_{12}N_{16.6}H_{10.2}Li_{1.9}Br_{1.3}$ determined using the C, N, H and Br contents found by combustion analysis, combined with Li required to satisfy charge balance. The signal intensity for the IF-PTI sample was approximately 100 times lower than this, indicating a stoichiometry $C_{12}N_{16.2}H_{13.3}Li_{0.015}Br_{0.05}$ when combined with the C, N, H, Br contents determined by combustion analysis.

JCPDS numbers

The JCPDS number for PTI·LiCl is PDF# 01-080-9035.

Hanawalt index of PTI·LiCl is included below, with the intensity being normalised to 999:

2θ	d(Å)	I	h	k	l
29.101	3.06599	999	1	0	2
26.385	3.37510	855	0	0	2
32.269	2.77188	353	2	1	0

None of the other carbon nitride materials detailed in this report have been assigned JCPDS numbers.

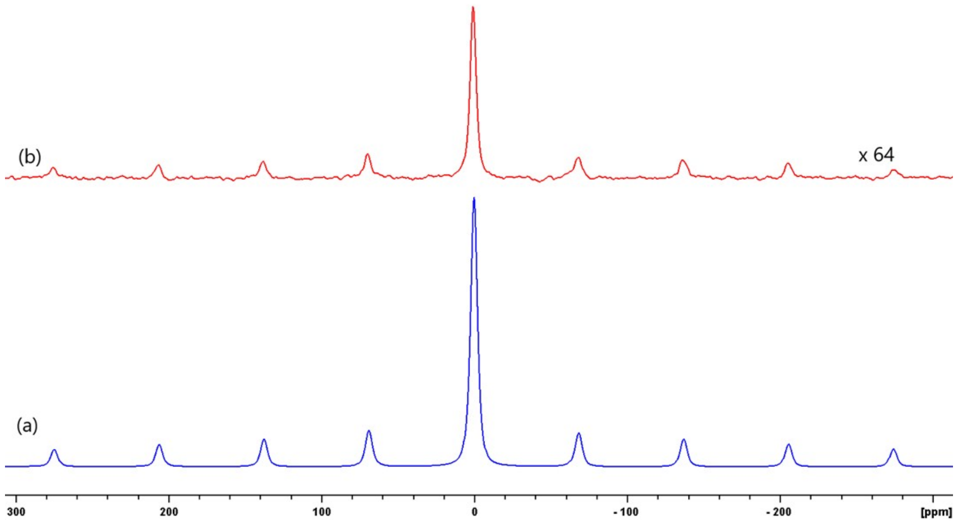

Figure S1. ^{7}Li MAS NMR spectra of (a) PTI·LiBr and (b) IF-PTI produced by washing PTI·LiBr Soxhlet extraction in deionized water. Both spectra were accumulated under the same conditions for 20 hours. Due to the significantly smaller lithium content, the intensity scale of the upper spectrum (b) has been expanded by a factor x64.

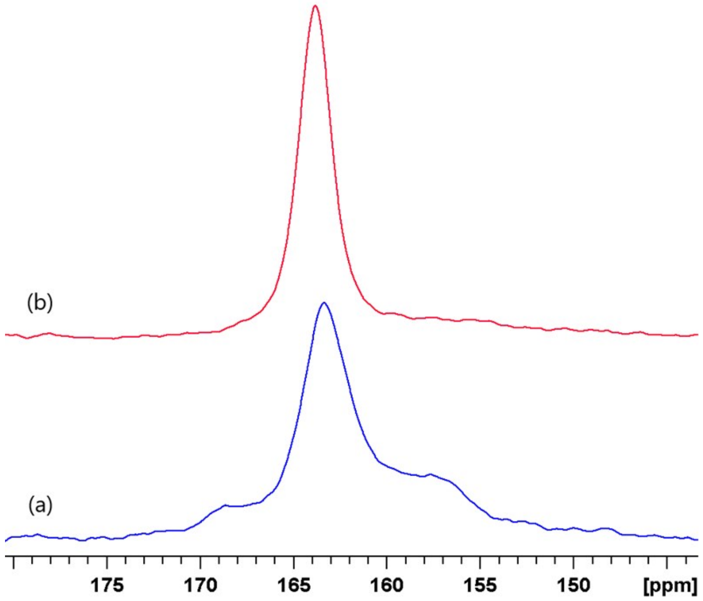

Figure S2. ^{13}C CP MAS NMR spectra of (a) PTI·LiBr and (b) IF-PTI following washing by Soxhlet extraction in deionized water. The chemical shift of the isotropic peak is 163.4±0.2 ppm (with "shoulder" peaks at 168±1 ppm and 158±1 ppm) in the spectrum of PTI·LiBr in (a) and 163.9±0.1 ppm in the spectrum of IF-PTI (b). The linewidths of the isotropic peak are 210±10 Hz (PTI·LiBr) and 120±6 Hz (IF-PTI).

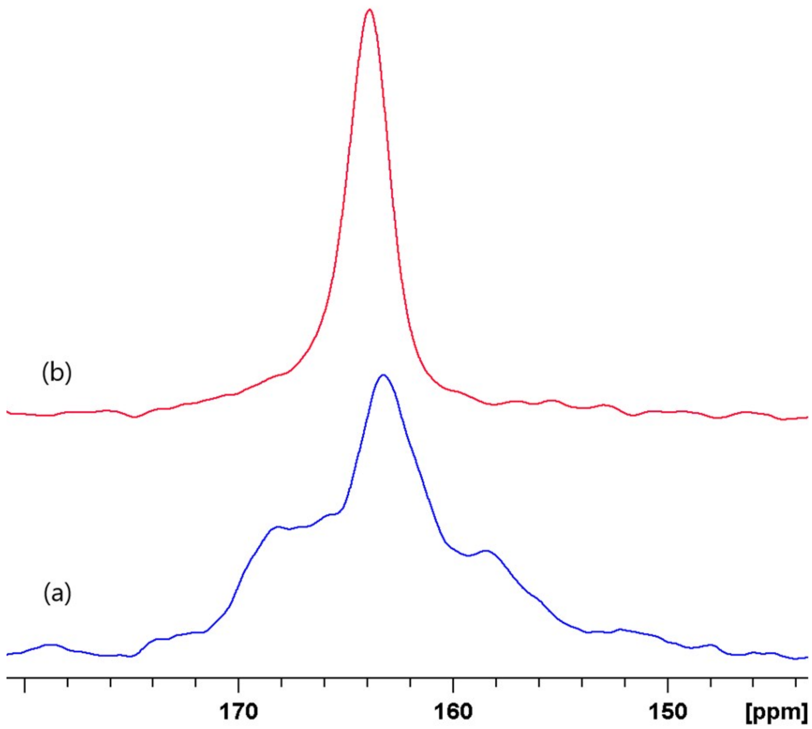

Figure S3. Single-pulse ^{13}C MAS NMR spectra of (a) PTI·LiBr and (b) IF-PTI. The chemical shift of the isotropic peak is 163.3±0.2 ppm (with “shoulder” peaks at 168±1 ppm and 158±1 ppm) in the spectrum of PTI·LiBr in (a) and 163.9±0.1 ppm in the spectrum of IF-PTI (b). The linewidths of the isotropic peak are 290±15 Hz (PTI·LiBr) and 100±5 Hz (IF-PTI).

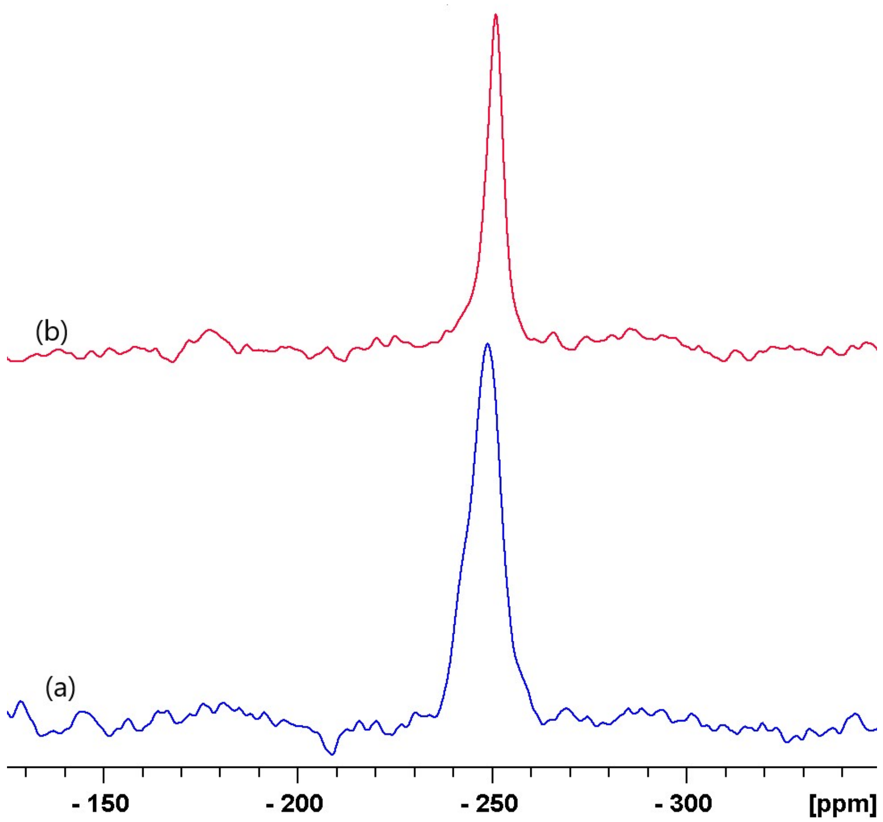

Figure S4. ^{15}N CP MAS NMR spectra of (a) PTI·LiBr and (b) IF-PTI. The chemical shift of the isotropic peak is -248.9±0.2 ppm, with a shoulder occurring at -244±1 ppm, in the spectrum of PTI·LiBr in (a) and -251.0±0.2 ppm for IF-PTI (b). The linewidth sof the isotropic peak are 210±10 Hz(PTI·LiBr) and 75±4 Hz (IF-PTI).

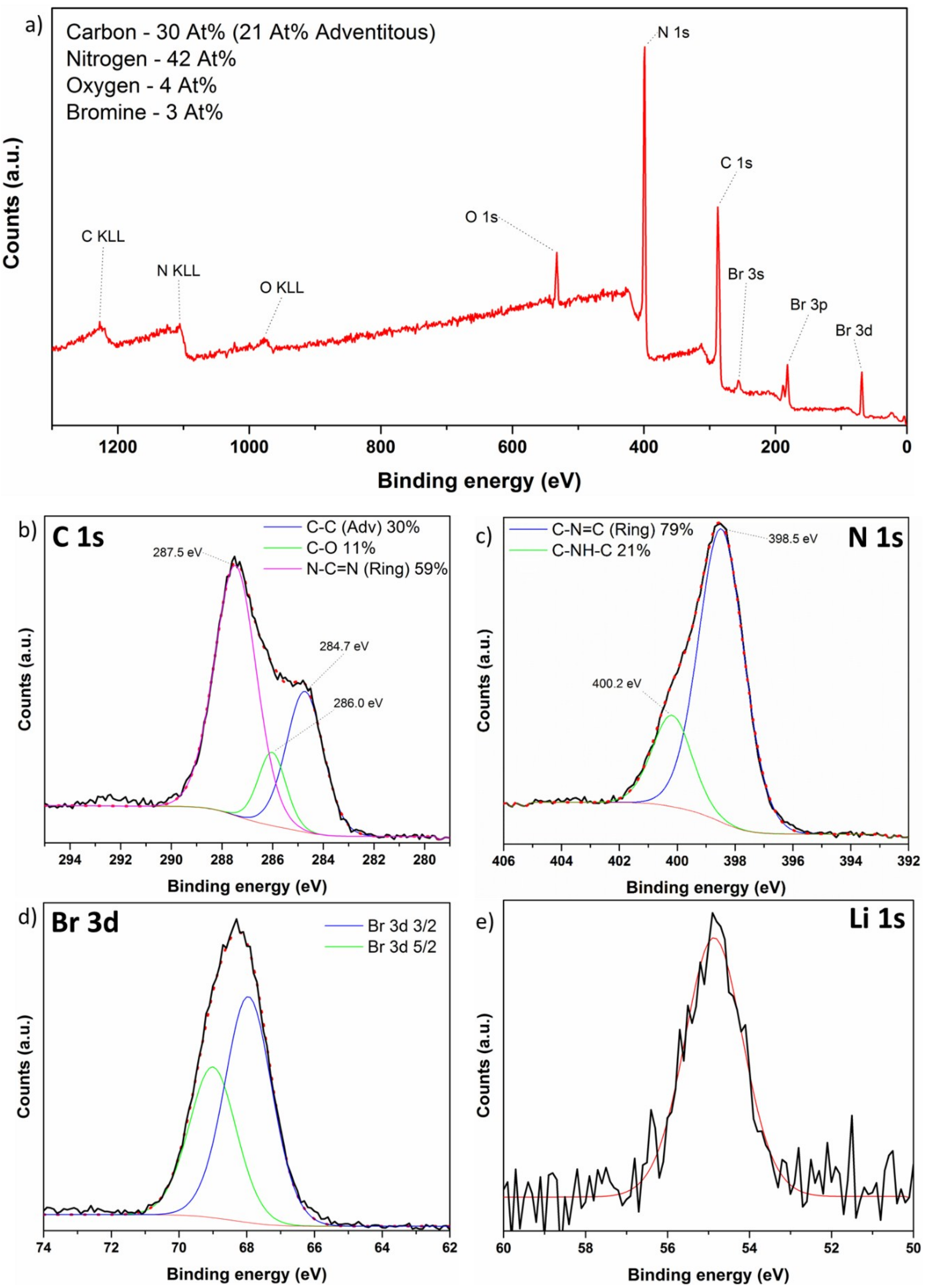

Figure S5. XPS of PTI-LiBr. (a) Survey spectrum. (b) Carbon 1s spectrum. (c) Nitrogen 1s spectrum. (d) Bromine 3d spectrum. (e) Lithium 1s spectrum. Atomic percentages of different elements are shown. The adventitious carbon content is indicated in brackets.

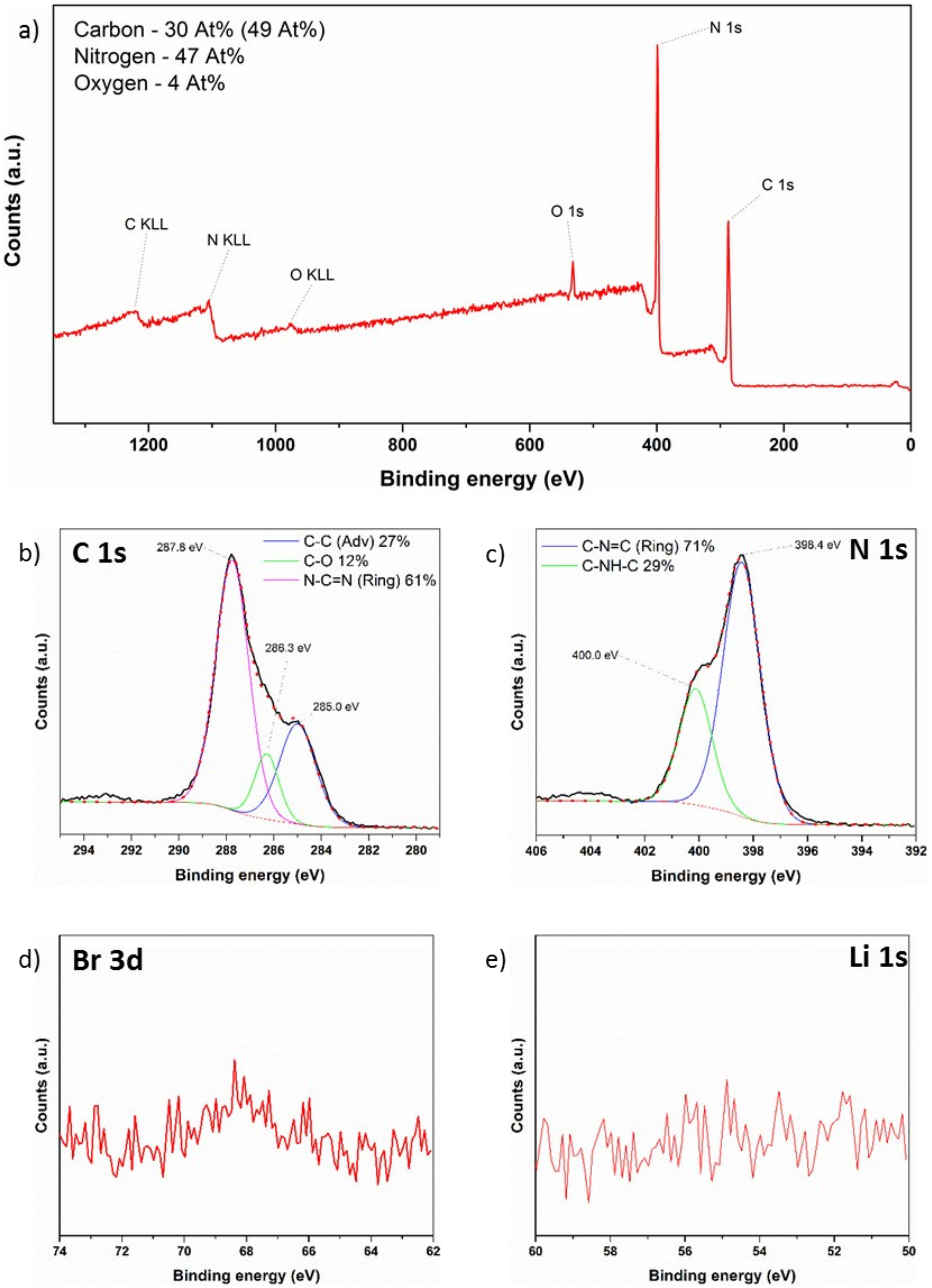

Figure S6. XPS of IF-PTI. (a) Survey spectrum. (b) Carbon 1s spectrum. (c) Nitrogen 1s spectrum. (d) Bromine 3d spectrum. (e) Lithium 1s spectrum. Atomic percentages of different elements are shown. The adventitious carbon content is indicated in brackets.

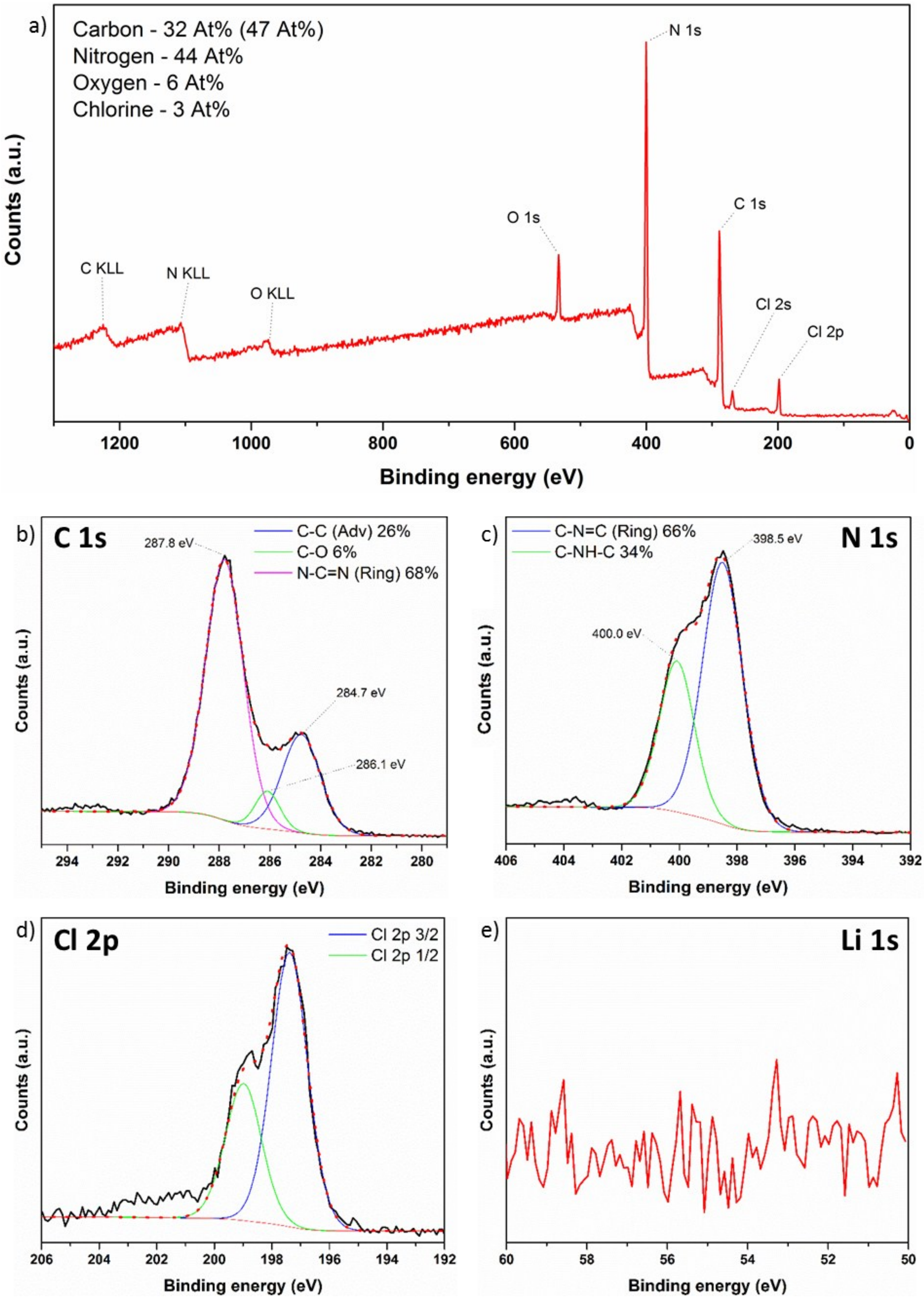

Figure S7. XPS of PTI·HCl. (a) Survey spectrum. (b) Carbon 1s spectrum. (c) Nitrogen 1s spectrum. (d) Chlorine 2p spectrum. (e) Lithium 1s spectrum. Atomic percentages of different elements are shown. The adventitious carbon content is indicated in brackets.

References:

1. M. J. Bojdys, J. O. Muller, M. Antonietti and A. Thomas, *Chem. Eur. J.*, 2008, **14**, 8177-8182.
2. E. J. McDermott, E. Wirnhier, W. Schnick, K. S. Virdi, C. Scheu, Y. Kauffmann, W. D. Kaplan, E. Z. Kurmaev and A. Moewes, *J. Phys. Chem. C*, 2013, **117**, 8806-8812.
3. J. E. Espidel, R. K. Harris and K. Wade, *Magn Reson Chem*, 1994, **32**, 166-172.
4. E. Wirnhier, M. Doblinger, D. Gunzelmann, J. Senker, B. V. Lotsch and W. Schnick, *Chem. Eur. J.*, 2011, **17**, 3213-3221.
5. S. Y. Chong, J. T. A. Jones, Y. Z. Khimyak, A. I. Cooper, A. Thomas, M. Antonietti and M. J. Bojdys, *J. Mater. Chem. A*, 2013, **1**, 1102-1107.
